# An active ingredient isolated from *Ganoderma lucidum* promotes burn wound healing via TRPV1/SMAD signaling

**DOI:** 10.18632/aging.204119

**Published:** 2022-06-13

**Authors:** Chunwei Jiao, Hao Yun, Huijia Liang, Xiaodong Lian, Shunxian Li, Jiaming Chen, Javeria Qadir, Burton B. Yang, Yizhen Xie

**Affiliations:** 1 Guangdong Yuewei Edible Fungi Technology Co., Ltd., Guangzhou 510663, P. R. China; 2 State Key Laboratory of Applied Microbiology Southern China, Guangdong Provincial Key Laboratory of Microbial Culture Collection and Application, Guangdong Open Laboratory of Applied Microbiology, Guangdong Institute of Microbiology, Guangdong Academy of Sciences, Guangzhou 510070, P. R. China; 3 Guangdong Yuewei Bioscience Co., Ltd., Zhaoqing 526000, P. R. China; 4 Sunnybrook Research Institute, Department of Laboratory Medicine and Pathobiology, University of Toronto, Toronto, ON M5S 1A8, Canada

**Keywords:** *Ganoderma lucidum* spore oil, wound healing, network pharmacology

## Abstract

The mushroom *Ganoderma lucidum* is a traditional Chinese medicine and *G. lucidum* spore oil (GLSO) is the lipid fraction isolated from *Ganoderma* spores. We examined the effect of GLSO on burn wound healing in mice. Following wounding, GLSO was applied on the wounds twice daily. Repair analysis was performed by Sirius-Red-staining at different time points. Cell proliferation and migration assays were performed to verify the effect of GLSO on growth. Network pharmacology analysis to identify possible targets was also carried out, followed by Western blotting, nuclear translocation, cell proliferation, and immunofluorescence assays for in-depth investigation of the mechanism. Our study showed that GLSO significantly promoted cell proliferation, and network pharmacology analysis suggested that GLSO might act through transient receptor potential vanilloid receptor 1 (TRPV1)/SMAD signaling. Furthermore, GLSO elevated SMAD2/3 expression in skin burn and promoted its nuclear translocation, and TRPV1 expression was also increased upon exposure to GLSO. Cell proliferation and immunofluorescence assays with TRPV1 inhibitor showed that GLSO accelerated skin burn wound healing through TRPV1 and SMADs signaling, which provides a foundation for clinical application of GLSO in the healing of deep skin burns.

## INTRODUCTION

A burn is one of the most severe injuries of the skin that can lead to chronic wounds and influence daily life [1, 2]. Treatment of a deep-degree burn is extremely important, and includes initial first aid, assessment of the area and extent of the burn, fluid resuscitation, wound excision, transplantation and coverage, as well as infection control and nutritional support [3, 4]. Anti-infection surroundings and proliferation are essential in burn injury healing [5, 6]. At present, anodyne, anti-infective drugs, and growth hormones are widely used in deep-burn medical treatment [7–9]. However, drugs that are capable of exerting both anti-infection and proliferation effects simultaneously, are rarely reported.

The mushroom *Ganoderma lucidum* (*G. lucidum Leyss. ex Fr.*) *Karst* (also known as Reishi or Lingzhi) is one of the most intriguing traditional Chinese medicines (TCMs) for more than 2000 years, and is documented by the Chinese Pharmacopoeia and Dietary Supplement Code of the United States Pharmacopoeia. In recent years, various research studies have considered the biological effects of *G. lucidum* sporoderm-broken spores with advancement in sporoderm-breaking technology. Our previous research has shown that *G. lucidum* spore oil (GLSO) has an anti-inflammatory effect on skin wound healing through skin microbiota regulation [10]. However, the effect of GLSO on proliferation in skin wound healing and the underlying mechanism is yet to be fully understood, thus, requiring further investigations.

Network pharmacology is a validated discipline with ability to elucidate complex pharmacological mechanisms of effective substances in various herbs and herbal pairs as well as the TCM ingredients by integrating bioinformatics, cheminformatics, and network biology [11–15]. Network pharmacology analysis has widely been used in the recent years. In the present study, we used network pharmacology and experimental verification combination method to reveal potential disease targets of GLSO in burns and the underlying mechanism of action for GLSO in the proliferation phase of skin wound healing.

## MATERIALS AND METHODS

### GLSO preparation

Spores were collected from the fruit body of *G. lucidum*, which grew in the Dabie Mountain of An-Hui Province, and treated with wall-breaking procedures at good-manufacturing-practice (GMP) manufacturing facilities of Guangdong Yuewei Edible Fungi Technology Co. Ltd., Guangzhou, China. The lipid fraction of the spores was isolated by an extremely high-pressure machine using liquid CO_2_. The main constituent compounds of GLSO were tested by high-performance liquid chromatography (HPLC) analysis. The methods were provided in our previous publications [10, 16].

### Animals and experiments

### Animals

All animal studies were conducted in accordance with the Guidelines for the Use and Care of Laboratory Animals of the National Institutes of Health and the Animal Welfare Act Regulations. Each *in vivo* experiment was approved by the Ethics Committee of Guangdong Institute of Microbiology. The mice (strain ICR mice) were purchased from the Jinan Pengyue Experimental Animal Breeding Company (Shandong, China). Male mice aged 7–8 weeks with body weight 32 ± 3 g, were used for the studies. Each mouse was maintained in a constant environment (20–22° C, 12-h light-dark cycle) with a normal diet, and individually housed in a cage to prevent further damage to the wounds. In this study, the experimental protocols were approved by the Committee of the Guangdong Institute of Microbiology Animal Center (Permit Number: SYXK(YUE)-2021-0156).

### Wound creation and treatments

The burn wound creation on the mouse skin and the procedure to handle the mice have been described by us, previously [10]. In brief, the mice were randomly categorized into three groups (n=9 for each group): sham group, control group, and GLSO treated. Mice were anesthetized by intraperitoneal injection and shaved at the middle back prior to introducing the burn wound. Mice in the sham group underwent shaving only. Mice in the control group underwent skin wounding, while those in the GLSO group received GLSO treatment upon the burn wound to test the effect of GLSO on wound repair. A homemade device consisting of a hollow rubber tube with 2 cm internal diameter was used to produce burn wound in the animal by placing it in direct contact with the skin in the shaved area. Following wounding with boiling water, 0.1 mL pure GLSO was applied on the wound twice daily for up to 5 days in the GLSO-treated group. The mice were, then, sacrificed by cervical dislocation at days 1, 3, and 5 (n=3 per group per day) to collect wounded skin and serum samples at these time points. Subsequently, each wounded skin sample was divided into three parts for further analyses.

### Tissue analysis

The tissues were subjected to Sirius Red staining. To do so, the collected tissue samples contained the unwounded normal skin as well as the wounded skin area to allow the identification of the wound edge for subsequent analysis. The tissues were fixed with formalin and cut into 3-μm sections for Sirius Red staining following an already established lab protocol [10]. Images were captured using a polarizing microscope (Nikon, Tokyo, Japan). The ratio of collagen I to collagen III was calculated using Image-pro Plus 6.0 (Media Cybernetics, MD, USA) by scanning the number of clearly stained pixels for the corresponding collagen-stained area.

To perform immunofluorescent staining, all tissues covering the normal unwounded skin area and the wounded area were fixed with formalin, embedded in paraffin, and cut into 3-μm sections. These sections were deparaffinized through a graded series of dimethylbenzene and ethanol. Antigens were retrieved by incubation in citric acid buffer (pH 6.0, ab93678, Abcam) at 100° C for 20 min. The sections were incubated with 3% H_2_O_2_ for 25 min to block endogenous peroxidase activity. Cell samples were fixed with 4% paraformaldehyde for 30 min and treated with permeabilizing agent 0.2% Triton X-100 (301G056, Solarbio, Beijing, China) for 10 min. The skin tissues and cell samples were incubated with 10% goat serum albumin at room temperature for 25 min to block non-specific binding. Subsequently, these sections were incubated with primary antibody to SMAD 2/3 (diluted in PBS, 1:200, CST, 8685s) and TRPV1 (diluted in PBS, 1:100, abs134462, Absin) at 4° C overnight. Following washing with PBS thrice, the sections were incubated with goat anti-rabbit immunoglobulin-G immunofluorescence antibody (diluted in PBS, 1:200, 4412S, Cell Signaling Technology, MA, USA) at room temperature for 50 min. The sections were washed again, and incubated with 4’,6-diamidino-2-phenylindole (DAPI, G1012, Servicebio, Wuhan, China) at room temperature for 5 min. Images were captured using an immunofluorescence inverted microscope (EVOS™ FL Auto 2 Imaging System, Shanghai, China).

### Cell activity assays

Human keratinocyte line HaCaT cells were purchased from the Deutsche Sammlung von Mikroorganismen und Zellkulturen (DSMZ, Braunschweig, Germany). The cells were cultured in Dulbecco’s modified eagle medium (DMEM) (C11095500BT, Gibco, NY, USA) supplemented with 10% fetal bovine serum (FBS, 10099-141, Gibco) at 37° C in a humidified 5% CO_2_ atmosphere.

In cell proliferation assay, HaCaT cells (1×10^4^ cells per well) were seeded in 24-well tissue culture plates in DMEM supplemented with 3% FBS for 24 h. Except for the control group, the cells were divided into four groups and treated with different GLSO concentrations: 20, 40, 60, and 80 μg/mL for 24, 48, and 72 h. At each time point, the cells were counted using a cell counter (IC 1000, Countstar, Shanghai, China). The optimal GLSO concentration for cell proliferation was determined in each of the four groups.

To test the effects of TRPV1 on cell proliferation, HaCaT cells (1×10^4^ cells per well) were plated and pre-incubated with TRPV1 inhibitor (0.0258 μg mL^-1^ SB705498, Absin, Shanghai, China) for 3 and 16 h. The cells were subsequently treated with 60 μg/ml GLSO for 48 h. Finally, the cells were divided into two parts; one part was fixed and observed under a standard optical microscope, followed by cell number determination, while the other part was used for immunofluorescence assay for TRPV1 and SMAD 2/3 signaling.

In cell migration assay, the *in vitro* culture scratch assay was adopted as a wound healing model to test the effect of GLSO on HaCaT migration/wound healing process. The cells (1×10^6^ cells per well) were grown in six-well tissue culture plates for 24 h. Following cell adherence, vertical and horizontal cross-shape scratches were created in each well by scraping the cell layer with a 10-μL pipette tip. The wells were washed three times with phosphate-buffered saline (PBS) to clear any detached cells, and divided into four groups. Apart from the control group, the cells were treated with different GLSO concentrations: 20, 60, and 80 μg per ml for 24, 48, 72, and 96 h. Cell migration in the scratch area was photographed and the closure rates of scratch area were calculated using Image-pro Plus 6.0.

### Network pharmacology analysis

We performed a network analysis to identify potential targets of GLSO. First, possible GLSO targets were searched using an extensive literature. Second, the two-dimensional (2D) molecular structures of the main compounds characterized by HPLC were searched on PubChem, and the molecular similarity match tool based on the simplified molecular input line entry specification (SMILES) in SwissTargetPrediction (*P*<0.05) was used to identify the potential proteins targeted by GLSO. Third, we used the keyword “burn” to search relative targets of burn in the Online Mendelian Inheritance in Man (OMIM) database and GeneCards. Fourth, an interaction network for the main compounds and putative GLSO targets for burn treatment was constructed based on their interaction data using protein-protein interaction (PPI) by STRING (Version 11.0), and visualized using Cytoscape software (Version 3.7.2, National Resource for Network Biology, USA). The degree, betweenness centrality, and closeness centrality were analyzed using the Network Analyzer plugin in Cytoscape, which were then applied to determine the topological importance of nodes in the network. Finally, the putative targets of GLSO were used in Kyoto Encyclopedia of Genes and Genomes (KEGG) analysis by Metascape to elucidate the important and related protein pathways.

### Western blot assay

For western blotting, frozen skin tissues were ground in liquid nitrogen and lysed with ice-cold Radio-immunoprecipitation Assay (RIPA) buffer (R0278, Sigma, Shanghai, China). The supernatant was collected after centrifugation of the tissue lysate at 12,000 rpm in a refrigerated centrifuge (5804R, Eppendorf, Shanghai, China) at 4° C for 10 min, followed by the measurement of protein concentrations using a BCA assay kit (Thermo Fisher Scientific, NY, USA). Protein samples (40 μg per lane) were separated by 10% sodium dodecyl sulfate-polyacrylamide gel electrophoresis (SDS-PAGE) and transferred to polyvinylidene fluoride membranes (Millipore Immobilon, Darmstadt, Germany). The membranes were blocked with 5% skimmed milk in Tris-buffered saline with 0.1% Tween-20 (TBST) at room temperature for 2 h, and incubated with primary antibodies diluted in PBS against SMAD2/3 (8685s, CST, Shanghai, China), p-SMAD2/3 (8828s, CST), and β-actin (ab52866, Abcam, Shanghai, China) at 4° C overnight. These membranes were, then, washed three times with TBST for 10 min each and incubated with horseradish peroxidase-conjugated secondary antibody (ab6721, Abcam) at room temperature for 1 h. Protein signals were detected using electro-chemiluminescence (ECL) reagent (Thermo Fisher Scientific).

### SMAD 2/3 nuclear importation on lipopolysaccharide (LPS) stimulation

HaCaT cells were stimulated with LPS to verify the mechanism of GLSO in accelerating skin wound healing by SMAD 2/3 signaling. HaCaT cells (1×10^4^ cells per well) were seeded in 24-well tissue culture plates for 24 h. Cells were divided into four groups; two of which were treated with 1 μg/ml LPS for 16 h. Subsequently, the GLSO (no pre-LPS) and GLSO+LPS groups were treated with 60 μg/ml GLSO. After 24 h, the cells were collected for immunofluorescence assay to examine SMAD 2/3 nuclear translocation in each group, as described below.

### Statistical analysis

The results were presented as the mean ± standard deviation (SD). Normal distribution and statistical comparisons between groups were determined by one-way analysis of variance and least-significant difference test using SPSS version 19.0 (IBM, NY, USA). For statistical testing, the variance between each group was defined using the probability value P and values of P < 0.05 were considered to be statistically significant.

## RESULTS

### GLSO accelerated collagen fiber regeneration in skin burn wounds

**Figure 1:**
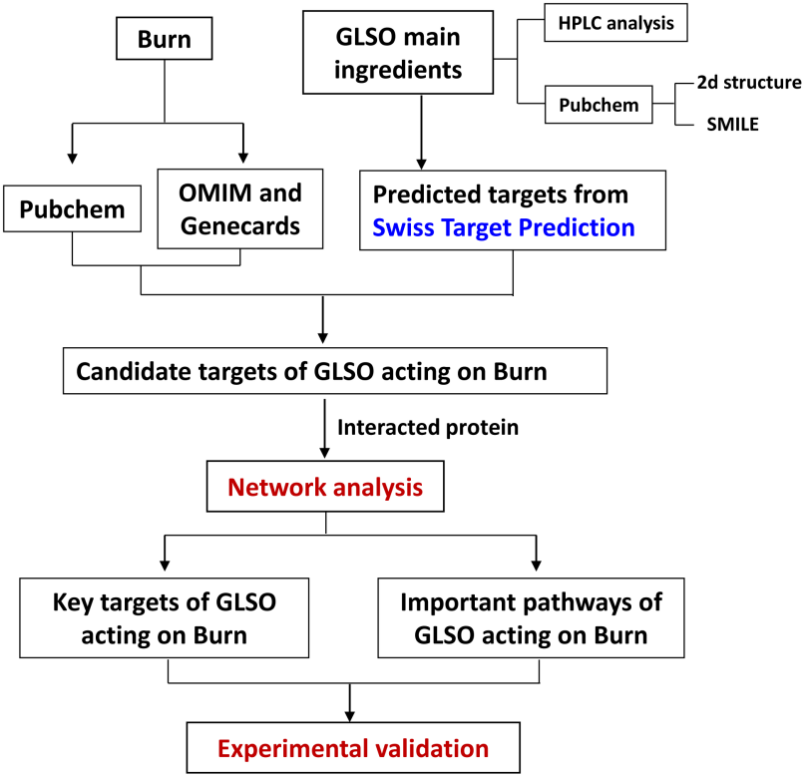
Workflow for GLSO treatment of skin burn injury.

**Figure 2:**
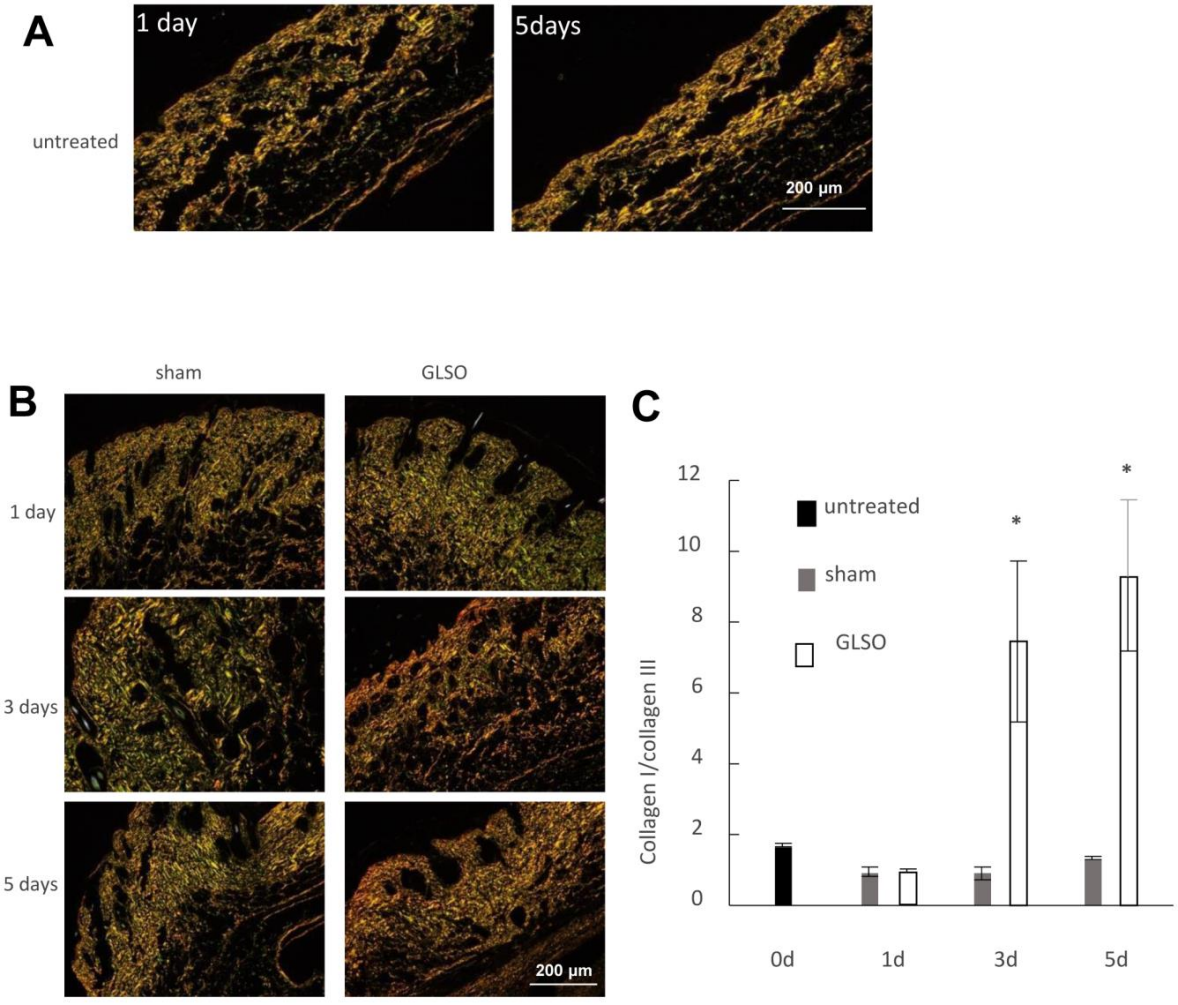
**The effect of GLSO on the ratio of collagen I to collagen III in skin burn in mice.** (**A**) Sirius Red staining of the sham mouse skin observed using a polarizing microscope. Scale bar=200 μm. (**B**) Sirius Red staining of the mouse skin treated with or without GLSO. Scale bar=200 μm. (**C**) Quantitative analysis of the ratio of collagen I to collagen III was performed (n=3 per group). The data are presented as the mean ± SD. ^*^P<0.05 versus sham.

In this study, we isolated the bioactive component of *Ganoderma lucidum* and examined its roles in promotion of burn wound repair. The procedure is shown in the diagram (Figure 1). Sirius Red staining was performed to determine the collagen I to III ratio in skin wounds treated with GLSO. Treated with picrosirius solution (0.1% Sirius Red in saturated aqueous picric acid, pH 2.0), the tissue sections would display collagen I as red-orange, while collagen III appearing as pale-green shades. As observed under a polarizing microscope, the Sirius Red stained sections showed little difference on day 1 and day 5 of the skin in the sham group (Figure 2A). In the GLSO treated group, there was more collagen I content (red area) than collagen III content (green area) in the GLSO group relative to the untreated (control) group (Figure 2B). Quantitative analysis of Sirius Red staining indicated that the ratio of collagen I to III in the GLSO group was significantly increased compared with the control group (Figure 2C).

### GLSO promoted HaCaT cell proliferation and migration

**Figure 3:**
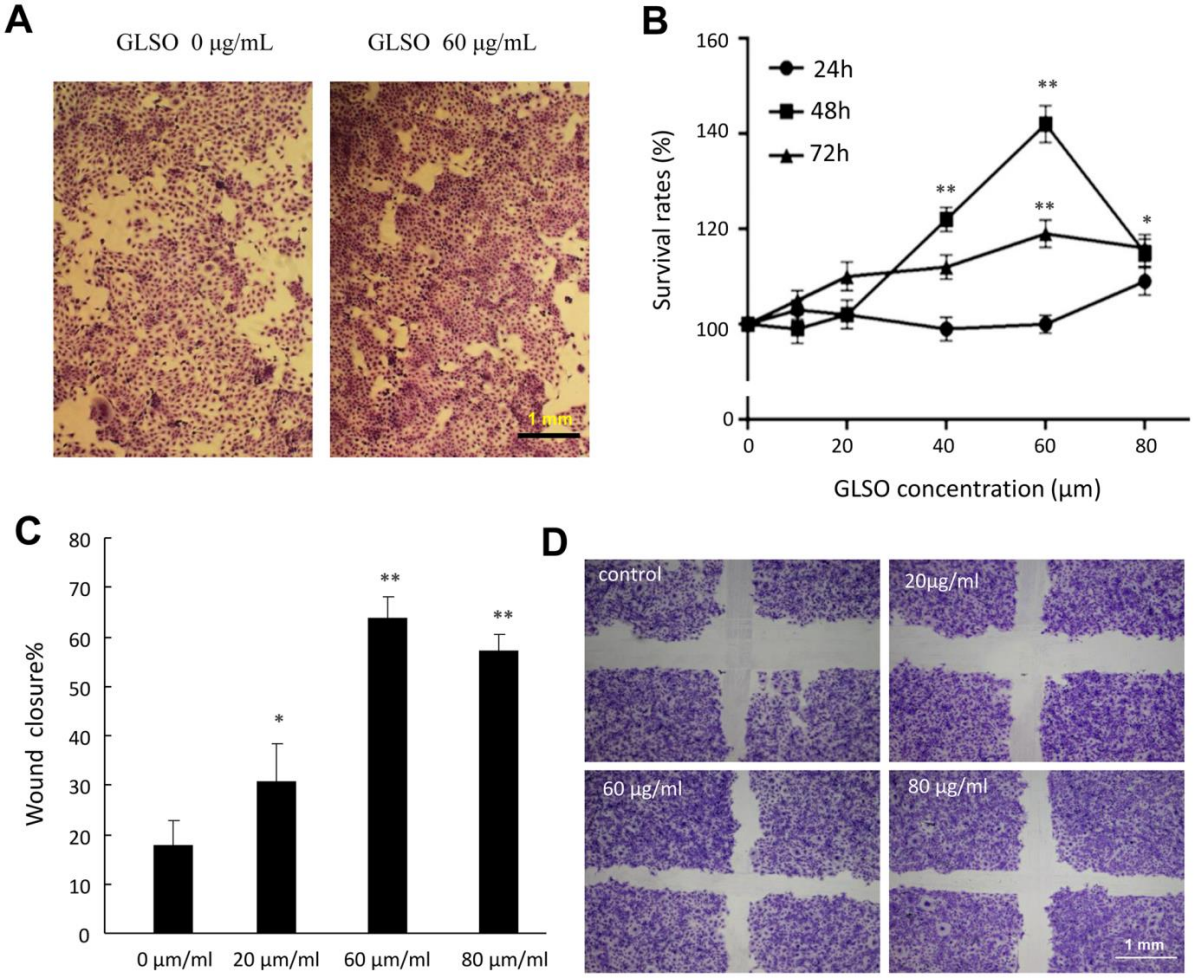
**The proliferation and migration effects of GLSO on HaCaT cells.** (**A**) Images of HaCaT cell growth with or without GLSO treatment. (**B**) Quantitative analysis of HaCaT cell proliferation with GLSO at different time points. (**C**) Images of HaCaT cell migration with or without GLSO treatment. Scale bar, 1 mm. (**D**) Quantitative analysis of wound closure by HaCaT cell migration with GLSO treatment. The data are presented as the mean ± SD. ^*^P<0.05, ^**^P<0.01 versus control.

HaCaT cells are human epidermal cells and are widely used to verify the effect of medicines. In the present study, we found that HaCaT cell proliferation was significantly increased 48 h after starting treatment of the wound with 60 μg/ml GLSO relative to the control group (Figure 3A, 3B). The cell migration assay also indicated that 60 μg/ml GLSO decreased the scratch distance and significantly increased the wound closure compared with the control group (Figure 3C, 3D).

### Network pharmacology analysis about GLSO on burn related targets

**Figure 4:**
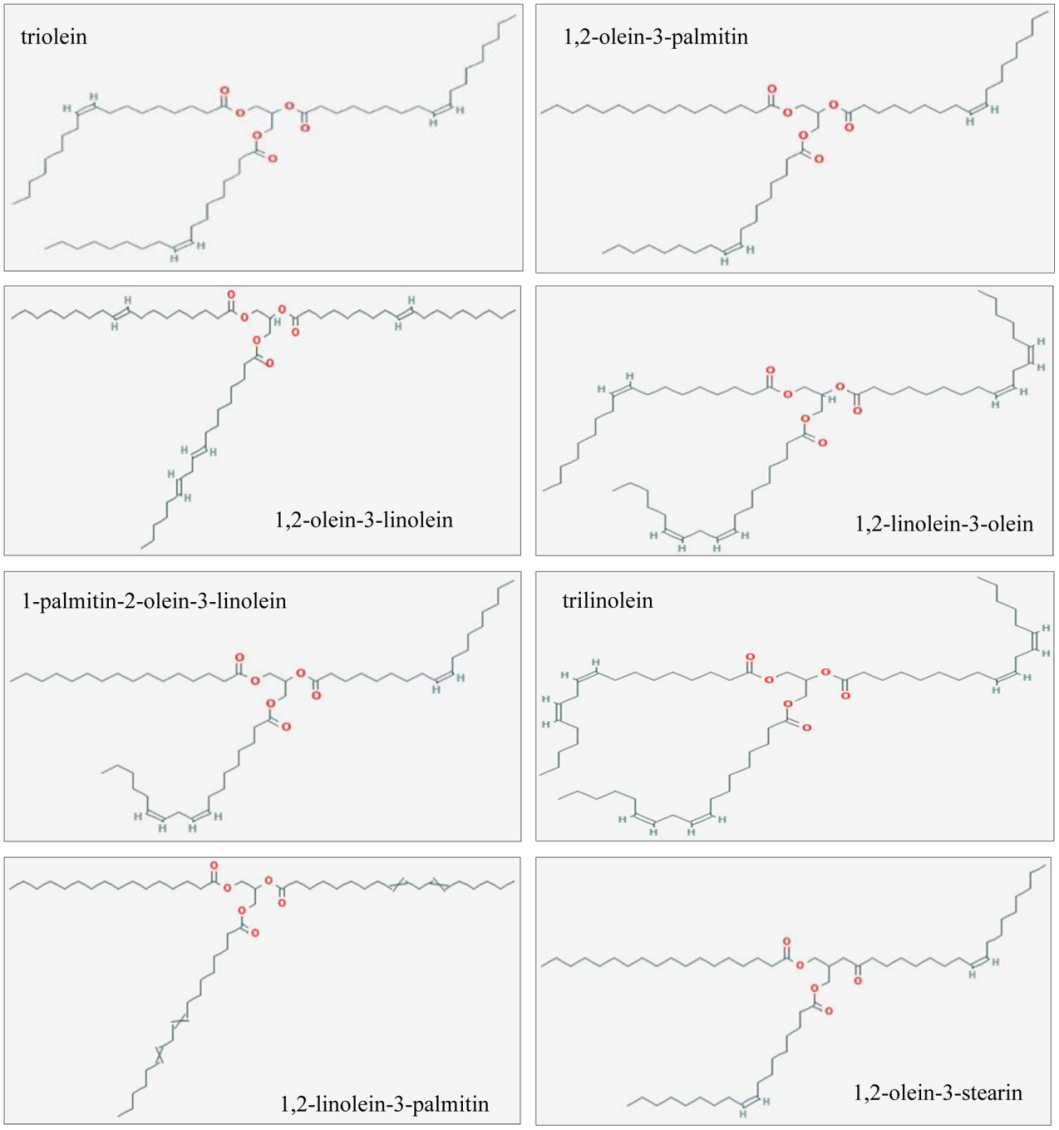
2D structures of the GLSO main ingredients.

**Figure 5:**
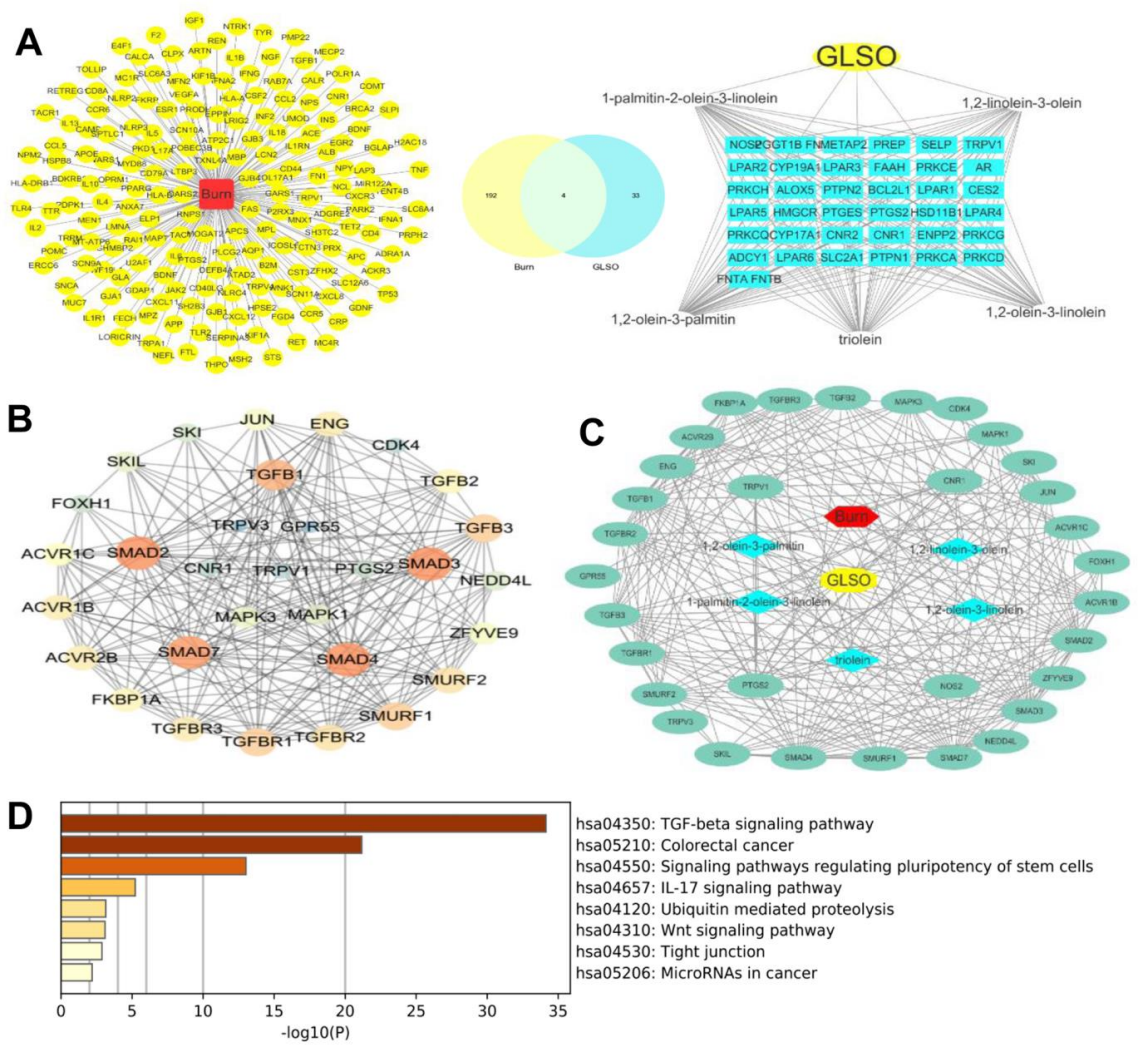
**Network pharmacology analysis of GLSO drug-ingredients’ targets.** (**A**) left, Burn disease-associated molecules; right, Potential targets of GLSO; middle, overlap of left and right. (**B**) Network analyzer analysis of GLSO drug-ingredients’ targets. (**C**) GLSO-ingredients-disease-targets network. (**D**) KEGG analysis of potential GLSO targets.

Trilinolein, 1,2-linolein-3-olein, 1,2-linolein-3-palmitin, 1,2-olein-3-linolein, 1-palmitin-2-olein-3-linolein, triolein, 1,2-olein-3-palmitin, and 1,2-olein-3-stearin are the main compounds of GLSO revealed by HPLC analysis. Their 2D structures are presented in Figure 4. The burn-associated molecules are shown in Figure 5A left panel and the potential GLSO targets are presented in Figure 5A right panel. Among these, no potential targets for trilinolein, 1,2-linolein-3-palmitin, or 1,2-olein-3-stearin were found in the SwissTargetPrediction. However, for the remaining five main GLSO components, 196 candidate targets related to burn injury were found from the database of OMIM and GeneCards. Four targets were found to be overlapping between them (Figure 5A middle), which were TRPV1, cannabinoid receptor 1 (CNR1), prostaglandin-endoperoxide synthase 2(PTGS2), and nitric oxide synthase 2 (NOS2). Regardingly, considerable amount of literature shows the transforming growth factor (TGF)-β signaling pathway to be important in skin wound healing [17]. With a PPI of four coincident targets and the TGF-β signaling pathway, we identified 31 targets as the putative targets of GLSO for burn disease treatment, and found that TRPV1 was an important protein among potential targets of GLSO related to burn disease. The results of the network dataset showed that SMAD2, SMAD3, SMAD4, SMAD7, and TGF-β1 were the most important proteins among the putative targets of GLSO. TGF-β3 was also important but had less influence than the aforementioned proteins (Figures 5B, 4C). Enrichment analysis of putative targets was performed and visualized by KEGG. The analysis showed that the TGF-β signaling pathway was the most important pathway for GLSO activity in burn treatment (Figure 5D). Comprehensively, the network analysis revealed that GLSO possibly improved burn wound healing by interacting with TRPV1 and the TGF-β signaling pathway.

### GLSO had a significant effect on SMAD2/3 and TRPV1 *in vitro* and *in vivo*

**Figure 6:**
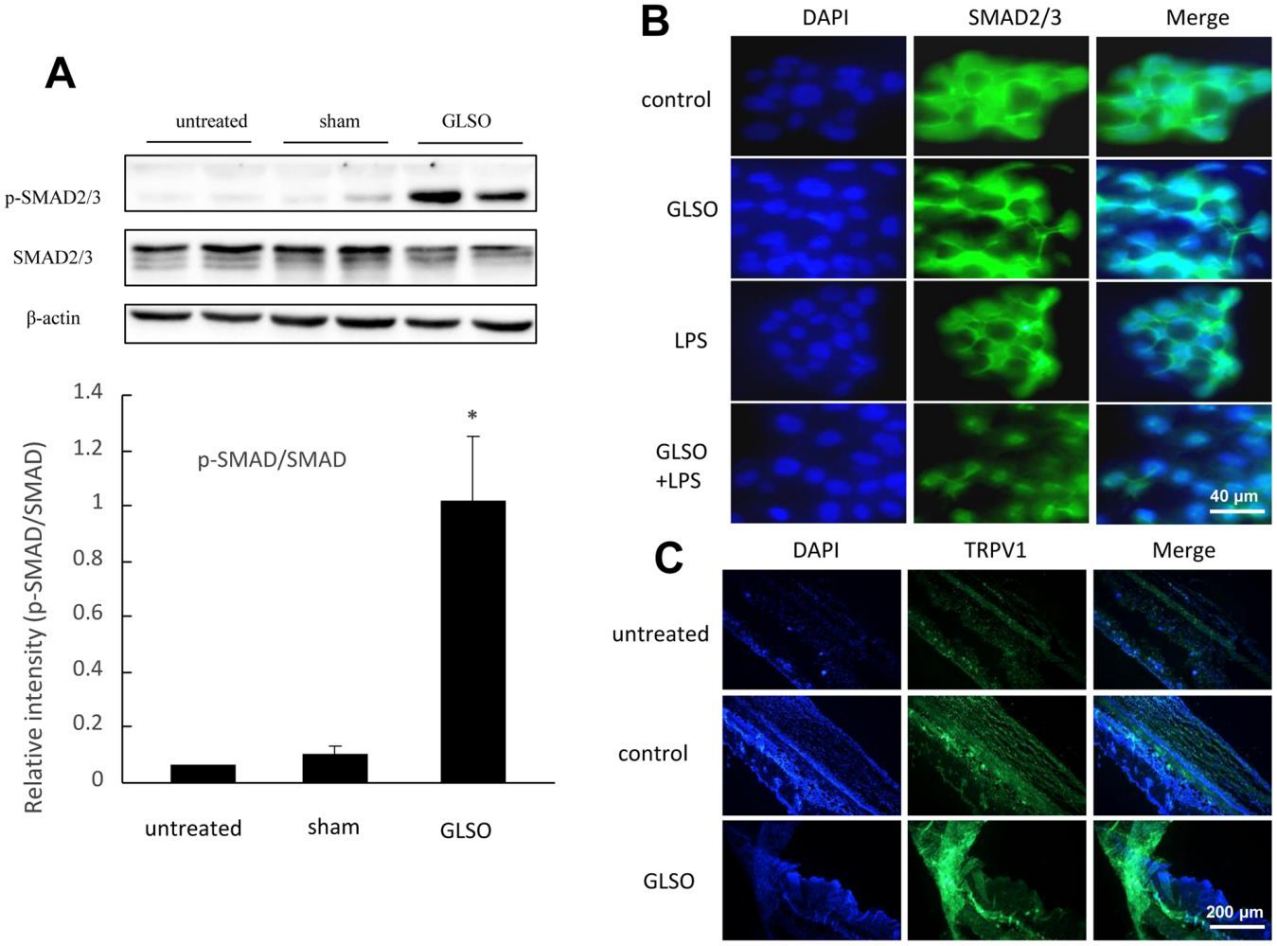
**Analysis of SMAD2/3 and TRPV1 expression in mouse skin burn.** (**A**) Western blot (upper) and quantitative analyses (lower) of SMAD2/3 and p-SMAD2/3. (**B**) Immunofluorescence staining of SMAD2/3 nuclear translocation on LPS (1 μg/mL) induction. Scale bar=200 μm. (**C**) Immunofluorescence staining of TRPV1 expression in skin burn upon GLSO treatment. Scale bar=200 μm. The data are presented as the mean ± SD. ^*^P<0.05 versus sham.

SMAD2 and SMAD3 were the most important proteins, characteristic of the TGF-β signaling pathway. Therefore, we examined SMAD2/3 and TRPV1 levels in the following experiments. Western blot analysis showed that SMAD2/3 phosphorylation levels were significantly increased, whereas SMAD2/3 expression levels were decreased upon GLSO treatment compared with the control group (Figure 6A). The results of immunofluorescence staining showed that GLSO clearly increased TRPV1 expression in skin burn wounds compared with the control group (Figure 6C). HaCaT cells stimulated with GLSO were used to simulate a burn model *in vitro*. GLSO accelerated SMAD2/3 nuclear translocation, and clearly promoted this translocation upon LPS stimulation (Figure 6B).

### GLSO promoted HaCaT cell proliferation by the interaction between SMAD2/3 and TRPV1 signaling

**Figure 7:**
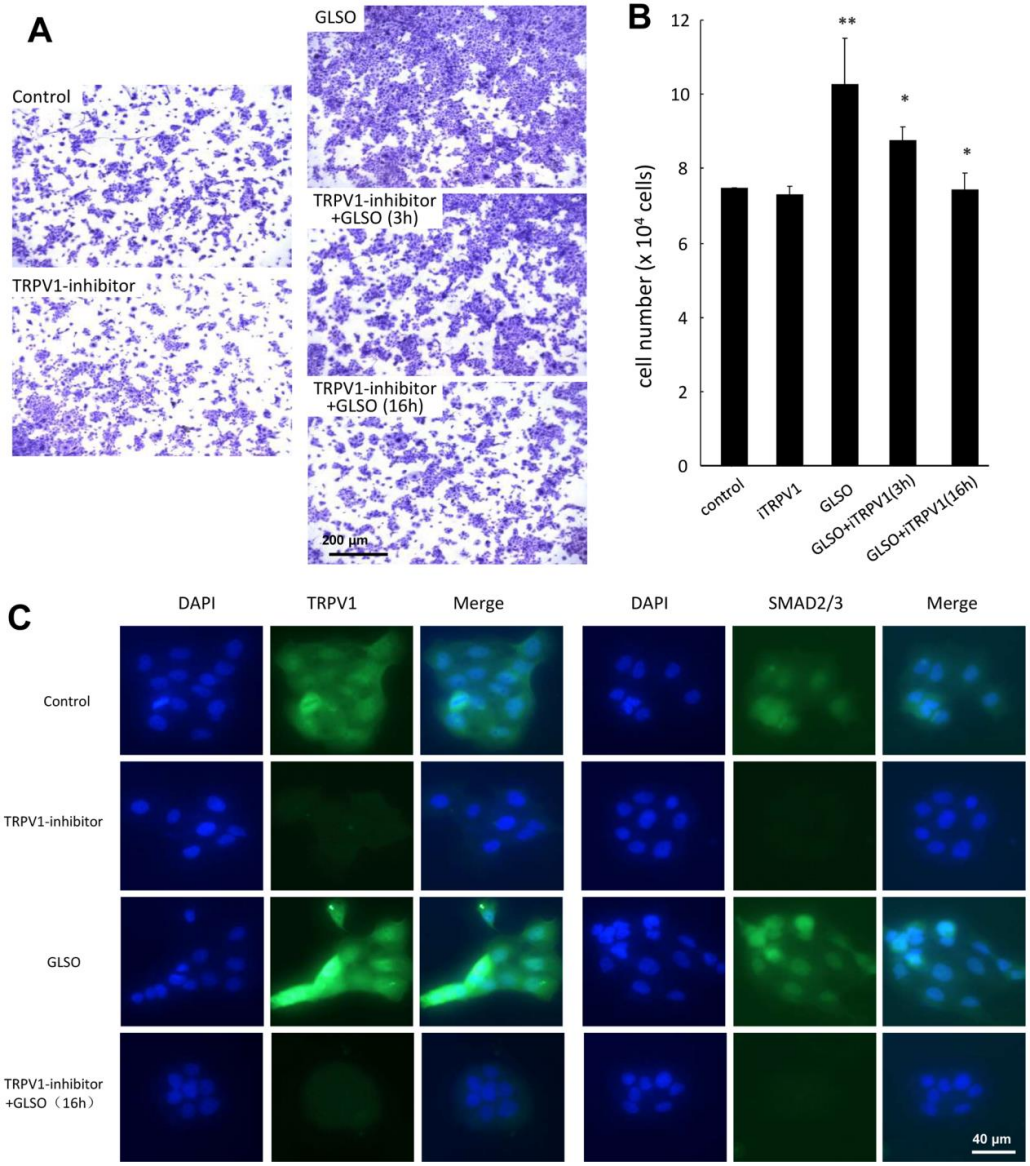
**Effect of TRPV1 inhibitor on HaCaT cell proliferation.** (**A**) HaCaT cells were pre-incubated with TRPV1 inhibitor (0.0258 μg/mL) for 3 and 16 h, followed by further incubation with GLSO for 48 h. GLSO-promoted cell proliferation was downregulated by TRPV1 inhibitor. (**B**) Quantitative analysis of reduced HaCaT cell numbers treated with TRPV1 inhibitor. (**C**) Immunofluorescence staining for TRPV1 and SMAD2/3 expression treated with TRPV1 inhibitor. Treatment with GLSO increased TRPV1 and SMAD2/3 expression that were down regulated by TRPV1 inhibitor. Scale bar=200 μm. The data are presented as the mean ± SD. *P<0.05, **P<0.01 versus control.

Finally, to verify whether TRPV1 influenced the skin burn wound healing by increasing SMAD2/3 expression, which were the most important signaling proteins in the TGF-β pathway, HaCaT cells were pre-incubated with TRVP1 inhibitor for 3 h or 16 h, and then incubated with GLSO for 48 h (Figure 7A, 7B). The GLSO enhancement of cell proliferation was significantly downregulated by the TRPV1 inhibitor. Immunofluorescence analysis showed that the addition of the TRPV1 inhibitor clearly decreased TRPV1 and SMAD2/3 expression compared with GLSO alone (Figure 7C).

## DISCUSSION

Hemostasis, inflammation, proliferation, and remodeling are the four main phases in skin wound healing. Large amount of literature showed that epidermal cells and cellular collagen fiber regeneration were important in epidermal and dermal healing [18–20]. In the present research, GLSO accelerated the regeneration of the stratum dermis. Collagen generation is considered to be an essential marker in wound repair, and the ratio of collagen I to collagen III represents the healing situation of collagen fibers in skin wound healing [21]. The reduction of collagen I during the stage of early skin wound repair would lead to poor granulation tissue formation and prolonged wound healing [22, 23]. GLSO increased the ratio of collagen I to collagen III, showing an important effect on collagen fiber regeneration in wound healing. Moreover, the proliferation and migration effects of GLSO on HaCaT cells showed that GLSO has the ability to promote regeneration of epidermal cells and accelerate the contraction of injured epidermis. Therefore, GLSO was possibly able to accelerate skin burn wound healing by promoting skin cell proliferation and migration during the proliferation stage.

Proliferation has a critical role in skin wound healing [24, 25]. Inflammatory cytokines induced by skin injury could initiate the proliferation stage and lead to the secretion of growth factors, including fibroblast growth factor, vascular endothelial growth factor, endothelial growth factor, and TGF [25–28]. Among these, TGF-βs are important influencing factors in modeling fibrotic diseases [29]. They are components of the TGF-β superfamily, which includes TGF-β1, TGF-β2, and TGF-β3 [30]. TGF-βs are multifunctional growth factors that can regulate cell proliferation, migration and differentiation, extracellular matrix deposition, and immune function [31]. TGF-βs are secreted by various cell types, including fibroblasts and macrophages. Considerable literature showed that the TGF-β signaling pathway is widely known to play crucial roles in proliferation and migration in the process of skin wound healing [17]. It has been shown that inflammatory cytokines can also activate the TGF-β signaling pathway [32]. They initiate the TGF-β/SMAD signaling pathway at the beginning of wound healing via TGF-βII receptors or SMADs family of proteins [33]. At present, the study of TGF-β/SMAD signal transduction is considered as the theoretical basis for investigating clinical wound healing and pathological research [34]. TGF-β/SMAD exert proliferation effects by binding to TGF-β receptors I and II [35], leading to SMAD2/3 phosphorylation. SMAD2/3 plays a key role as the hub of TGF-β signal transmission, characterizing the expression of TGF-β signaling [36]. GLSO promoted SMAD2/3 phosphorylation and increased the ratio of phosphorylated SMAD2/3 to SMAD2/3 in skin burn on mice, which showed that GLSO had the ability to activate SMAD2/3 and initiate the TGF-β/SMAD signaling pathway. The activation effect of GLSO on SMAD2/3 demonstrated that it has the ability to accelerate the proliferation stage in skin wound healing. *In vitro*, SMAD2/3 nuclear importation indicated that the proliferation effect on skin wound healing was initiated after the skin injury [37]. GLSO promoted SMAD2/3 nuclear transportation in HaCaT cells, and particularly more rapidly induced by LPS, showing that GLSO probably accelerated the progression from the inflammatory stage to the proliferation stage. Therefore, GLSO was able to accelerate skin wound healing by possibly activating the TGF-β/SMAD signaling pathway to promote proliferation in burnt skin.

Studies in recent decades have found that network pharmacology was suitable for examining the mechanisms of TCM, which had the features of being multi-components, multi-targets, and multi-pathways [38]. The results of HPLC analysis showed eight main ingredients, and their 2D structures were generated by PubChem. From the analysis of the main ingredients’ targets and burn disease targets, it was found that TRPV1, CNR1, NOS2, and PTGS2 were potential targets of GLSO in skin burn wound healing. TRPV1 was the most widely distributed transient receptor potential channel protein (TRP) in humans in the current study. This channel was readily activated by a variety of conditions, including temperature, pH, capsaicin, and adenosine triphosphate, both *in vivo* and *in vitro* [39]. Previous studies demonstrated that TRPV1 played a significant role in the inflammation intensity, pain transmission, and the treatment of related diseases, including that of the respiratory and nervous systems [40]. It was found that TRPV1 influenced various physiological activities, including cell proliferation and apoptosis, by mediating changes in the intracellular Ca^2+^ concentration [40]. TRPV1 is also a receptor for capsaicin, which is distributed in immune cells, organ epithelial cells, and keratinocytes, and mediates various physiological and pathological activities, including apoptosis, differentiation, and inflammation [41, 42]. Furthermore, CNR1 is downstream of TRPV1 [43]. NOS2 is a synthase for protein catalytic reaction [44, 45], and PTGS2 is a marker of cancer [45]. From the PPI with respect to the four potential targets of GLSO and the proliferation pathway TGF-β/SMAD signaling, it was found that TRPV1 was able to activate TGF-β/SMAD signaling via mitogen-activated protein kinase (MAPK) signaling, which regulated the energy metabolism. Comparably, NOS2, PTGS2, and CNR1 had no obvious direct association with TGF-β/SMAD signaling in the PPI analysis. The result of the network analyzer on Cytoscape showed that in the TRPV1/TGF-β signaling pathway, SMAD2 and SMAD3 were key targets and important proteins. KEGG analysis also indicated that the most important signaling pathway of all the above target proteins was TGF-β/SMAD signaling, which was consistent with the literature search results. From the results of network pharmacology, it was found that TRPV1 was the potential protein of GLSO to activate the TGF-β/SMAD signaling pathway.

A previous study indicated that TRPV1 knock-out led to impaired corneal healing in mice, and TGF-β signaling was clearly inhibited, as obviously influenced by TRPV1 [46]. Interestingly, it was also found that GLSO increased TRPV1 expression in burnt skin in the present research. To investigate further, TRPV1 inhibitor was used to arrest HaCaT cell proliferation to demonstrate the interaction between TRPV1 and TGF-β signaling. With the application of the TRPV1 inhibitor, the proliferation effect of GLSO on HaCaT cells was significantly inhibited, and TRPV1 and SMAD2/3 expression levels were decreased. Our results indicated that GLSO increased the proliferation effect on skin wound healing via TRPV1/SMAD signaling.

## CONCLUSIONS

In summary, GLSO improved burn healing by stimulating cell proliferation of skin wounds. In tissues, GLSO was observed to potentially elevate TRPV1 expression, initiated TGF-β/SMAD signaling more rapidly, and promoted SMAD2/3 nuclear translocation, thereby, accelerating the progress from the inflammatory stage to the proliferation stage in skin wound healing.
